# Sirtuin 5 regulates the proliferation, invasion and migration of prostate cancer cells through acetyl‐CoA acetyltransferase 1

**DOI:** 10.1111/jcmm.16016

**Published:** 2020-10-26

**Authors:** Jingqian Guan, Xizi Jiang, Junda Gai, Xiaodan Sun, Jinming Zhao, Ji Li, Yizhuo Li, Ming Cheng, Tengjiao Du, Lin Fu, Qingchang Li

**Affiliations:** ^1^ Department of Pathology College of Basic Medical Sciences China Medical University Shenyang China; ^2^ Department of Pathology The First Hospital of China Medical University Shenyang China; ^3^ Jilin Cancer Hospital Changchun China

**Keywords:** ACAT1, Gleason score, MAPK signalling pathway, prostate cancer, Sirtuin 5

## Abstract

Sirtuin 5 (SIRT5) is a NAD^+^‐dependent class III protein deacetylase, and its role in prostate cancer has not yet been reported. Therefore, to explore the diagnosis and treatment of prostate cancer, we investigated the effect of SIRT5 on prostate cancer. Sirtuin 5 was assessed by immunohistochemistry in 57 normal and cancerous prostate tissues. We found that the tissue expression levels of SIRT5 in patients with Gleason scores ≥7 were significantly different from those in patients with Gleason scores <7 (*P* < .05, R > 0). Further, mass spectrometry and pathway screening experiments showed that SIRT5 regulated the activity of the mitogen‐activated protein kinase (MAPK) pathway, which in turn modulated the expression of MMP9 and cyclin D1. Being a substrate of SIRT5, acetyl‐CoA acetyltransferase 1 (ACAT1) was regulated by SIRT5. SIRT5 also regulated MAPK pathway activity through ACAT1. These results revealed that SIRT5 promoted the activity of the MAPK pathway through ACAT1, increasing the ability of prostate cancer cells to proliferate, migrate and invade. Overall, these results indicate that SIRT5 expression is closely associated with prostate cancer progression. Understanding the underlying mechanism may provide new targets and methods for the diagnosis and treatment of the disease.

## 1 INTRODUCTION

Prostate cancer is the most common non‐skin cancer in men worldwide,^1^ and its diagnosis and treatment are very important. Although localized prostate cancer has a high long‐term survival rate, metastatic prostate cancer remains incurable.^2^ Early diagnosis and classification of prostate cancer are particularly important for its treatment.^3^

As early as 1992, Gleason proposed a scoring system for determining the malignancy of prostate cancer, which is currently the system most commonly used by pathologists and is a suitable grading indicator.^4^, ^5^, ^6^, ^7^ Using the Gleason system and with an enhanced understanding of the aetiology of prostate cancer, an increasing number of hormonal drug treatments,^8^, ^9^ immunotherapeutics^10^, ^11^ and surgical treatment methods^12^, ^13^ have been applied in prostate cancer. However, despite the available treatments, prostate cancer remains a major medical issue in men. Thus, developing new treatment methods and identifying targets for prostate cancer require further research.

Sirtuins belong to a highly conserved family of proteins. Mammals have seven sirtuins (SIRT1‐7) that regulate different metabolic and stress response pathways.^14^ SIRT5 plays a vital role in glycolysis, the tricarboxylic acid cycle, fatty acid oxidation, nitrogen metabolism, the pentose phosphate and antioxidant pathways, and apoptosis.^15^, ^16^, ^17^ SIRT5 promotes the growth of human non‐small cell lung cancer,^18^ and patients with high SIRT5 expression have a poor prognosis.^19^ In addition, SIRT5 is associated with liver,^20^ ovarian^21^ and breast^22^ cancers. However, its role in prostate cancer has not been reported to date.

Acetyl‐CoA acetyltransferase 1 (ACAT1) is a possible anticancer target.^23^ Its expression can be a potential prognostic marker for prostate cancer,^24^ and it may function as a promoter of this disease.^25^ This study aimed to investigate the role of the SIRT5‐ACAT1 axis in prostate cancer and identified new targets for the diagnosis and treatment of this disease.

## 2 MATERIALS AND METHODS

### 2.1 Patients and specimens

The prostate cancer specimens examined in this study were all obtained from the First Affiliated Hospital of China Medical University and were diagnosed and strictly Gleason scored by the Department of Pathology from 2013 to 2017. This study was approved by the Medical Research Ethics Committee of China Medical University, and informed consent was obtained from all patients.

### 2.2 Cell culture

All cell lines were obtained from the Shanghai Cell Bank (Shanghai, China) and cultured in RPMI 1640 medium with foetal bovine serum (FB15015; Clark Biosciences, Richmond, VA, USA). LNCaP and PC‐3 cells were cultured according to the manufacturer's instructions in medium containing 10% foetal bovine serum (FBS) with no antibiotics and maintained in a 5% CO_2_ incubator at 37°C.

### 2.3 Plasmid construction and transfection

SIRT5 siRNA (siG000023408B‐1‐5), ACAT1 siRNA (siB170726094953‐1‐5) and controls were purchased from Ruibo Biosciences (Guangzhou, China). Flag‐SIRT5 (RC200189) is an SIRT5 up‐regulated plasmid, and its control is Flag‐pCMV6; both were procured from Origene (Rockville, MD, USA). ACAT1‐3Flag, the overexpression plasmid of ACAT1, and its control, GV141, were obtained from GENE (Shanghai, China). Cells were transfected using Lipofectamine 3000 reagent (Invitrogen, Carlsbad, CA, USA) according to the manufacturer's instructions.

### 2.4 Immunohistochemistry and Gleason scores

Tissue sections were incubated with an SIRT5 rabbit polyclonal antibody (15122‐1‐AP, 1:500 dilution, ProteinTech Group, Rosemont, IL, USA) overnight at 4°C. The secondary antibody was added for 2 hours at 37°C. Nuclei were stained with haematoxylin for 10 minutes, and the expression of SIRT5 protein was observed under a microscope. Next, the tissue sections were scored strictly according to the latest Gleason scoring system. The chi‐square test was used to determine correlations between SIRT5 expression and clinicopathological characteristics. *P* < .05 was considered to represent a statistically significant difference.

### 2.5 Western blotting

Lysis buffer (P0013; Beyotime Biosciences, Shanghai, China) was used to extract total cell protein. We then added a mixture of protease and phosphatase inhibitors (B14002 and B15002; Biotool, Shanghai, China) at four times the volume of the cell pellet. Proteins (35 µg) were separated by SDS‐PAGE and transferred to a polyvinylidene difluoride membrane (EMD‐Millipore, Billerica, MA, USA). The membrane was blocked with 5% skim milk (232100; BD Biosciences, Franklin Lakes, NJ, USA) for at least 2 hours and then incubated with the appropriate primary antibody at 4°C overnight. It was then treated with the peroxidase‐conjugated secondary antibody for 1 hour at 37°C. The following primary antibodies were used: anti‐SIRT5 (15122‐1‐AP, 1:1000 dilution), anti‐MMP9 (10375‐2‐AP, 1:1000 dilution), anti‐cyclin D1 (60186‐1‐Ig, 1:1000 dilution) and anti‐ACAT1 (16215‐AP‐1, 1:1000 dilution) from ProteinTech Group; anti‐p44/42 mitogen‐activated protein kinase (MAPK) (extracellular signal‐regulated kinase (Erk)1/2) (4695s, 1:1000 dilution) and anti‐phospho‐p44/42 MAPK (Erk1/2) (4370s, 1:1000 dilution) from Cell Signaling Technology (Danvers, MA, USA); anti‐glyceraldehyde 3‐phosphate dehydrogenase (TA319654, 1:1000 dilution) from Origene; and mouse monoclonal anti‐ACAT1 (ab110290, 1:1000 dilution) from Abcam (Cambridge, MA, USA). Proteins were visualized using enhanced chemiluminescence (34080; Thermo Fisher Scientific, Waltham, MA, USA). ImageJ software (National Institutes of Health, Bethesda, MD, USA) was used to evaluate each band quantitatively, and values were compared using Prism 5.0 software (GraphPad, La Jolla, CA, USA).

### 2.6 Quantitative real‐time polymerase chain reaction (qRT‐PCR)

qRT‐PCR was performed with SYBR Green PCR Master Mix in a 7900HT Fast Real‐Time PCR System (Applied Biosystems, Foster City, CA, USA), in a total volume of 20 μL. The qRT‐PCR conditions were as follows: 95°C for 30 seconds, 40 cycles at 95°C for 5 seconds and 60°C for 30 seconds. The dissociation step was used to generate a melting curve and confirm amplification specificity. Gene expression relative to β‐actin was calculated using the 2^−ΔΔCt^ method.

### 2.7 MTS proliferation assay

Cells with up‐ or down‐regulated SIRT5 expression (3000 cells/well) were seeded into 96‐well plates and cultured in 10% FBS. Twenty microlitres of a 3‐(4,5‐dimethylthiazol‐2‐yl)‐5‐(3‐carboxymethoxyphenyl)‐2‐(4‐sulphophenyl)‐2H‐tetrazole salt (MTS) solution was added to test cell viability. After 1 hour of incubation at 37°C in the dark, the colour intensity of each plate was measured using a microplate reader at 490 nm (G3580, Promega, Madison, WI, USA). Five similar 96‐well culture plates of cells were prepared according to this method, and one plate was selected for MTS testing once a day.

### 2.8 Colony formation assay

Cells with altered expression of SIRT5 protein were inoculated into 6‐well culture plates at a density of 800 cells per well and cultured for 10‐14 days. The cells were then washed with phosphate‐buffered saline (PBS), fixed with pre‐cooled methanol, washed three times with PBS and finally stained with crystal violet. After drying, the colonies were counted manually.

### 2.9 Transwell invasion analysis

Prostate cancer cells (10^5^ cells per 100 μL) with increased or decreased SIRT5 protein expression were inoculated into Transwell chambers (8 μm pore size, Corning, NY, USA) for 24 hours to observe their migration ability. Cells that crossed the membrane were stained with crystal violet, dried and counted manually.

### 2.10 Immunofluorescence

An appropriate number of cells was seeded in a 10‐cm Petri dish (801002, NEST, Hong Kong, China), cultured in 10% FBS, fixed with 4% paraformaldehyde for 15 minutes and treated with 0.1% Triton X‐100 for 10 minutes. This was followed by blocking with 3% bovine serum albumin for at least 2 hours. Next, the cells were incubated with the SIRT5 primary antibody or ACAT1 primary antibody at 4°C overnight. After removal of the primary antibody, the plate was washed three times with PBS and incubated with the secondary antibody at 37℃ for 2 hours in the dark. MitoSOX Red Mitochondrial Superoxide Indicator(M36008, Invitrogen, UK) used for mitochondria. Cells were stained with 4′,6‐diamidino‐2‐phenylindole for 10 minutes. Finally, a confocal microscope (FV3000, Olympus, Tokyo, Japan) was used to obtain random cell images.

### 2.11 Co‐immunoprecipitation (Co‐IP) assays

The desired cell line was plated in two 10‐cm cell culture dishes. After the cells grew to confluence, they were lysed and centrifuged at 12 000 rpm for 15 minutes at 4℃. Next, 60 μL of Protein A/G Sepharose (P2012; Beyotime Biosciences) was added to the supernatant and stirred for at least 2 hours. The mixture was then centrifuged at 4°C and 1000 rpm for 5 minutes, following which the supernatant was divided into two parts. To one part was added the target antibody (8 µg), and to the other part, anti‐mouse/rabbit IgG (1:2000; ZSGB‐BIO, Beijing, China). The mixture was shaken overnight at 4°C in a chromatography cabinet. The following day, 25 μL of agarose A/G magnetic beads was added to each tube and incubated at 4°C for 6 hours. Next, the cell lysate was washed, heated in boiling water for 10 minutes and immunoblotted.

### 2.12 Active oxygen detection assays

The level of reactive oxygen species (ROS) in prostate cancer cells was determined using the probe 2',7'‐dichlorodihydroflfluorescein to detect diacetate (DCFH‐DA, Beyotime Biosciences), which when combined with intracellular oxygen forms dichlorofluorescein, a highly fluorescent compound. After 48 hours of transfection with SIRT5 plasmid or siRNA, the cells were cultured with 10 µmol\L DCFH‐DA in the dark for 20 minutes in a humidified atmosphere with 5% CO_2_ at 37°C. The cells were washed three times with cold PBS to remove excess fluorescent probes, counted and added to 96‐well plates (10^4^ cells/well). Absorbance was measured at 488 nm.

### 2.13 Inhibitor treatments

Cells were treated with the following: SIRT5 inhibitor 1 (HY‐112634, MCE, New Jersey, USA), a specific inhibitor of SIRT5, at a concentration of 0.1 μmol\L for 6 hours; K‐604 dihydrochloride (HY‐100400A, MCE), a specific inhibitor of ACAT1, at a concentration of 0.4 μmol\L for 6 hours; or MG‐132 (HY‐13259, MCE), a proteasome inhibitor that can effectively prevent hydrolysis of the 26S proteasome, at a concentration of 18.5 μmol\L for 24 hours.

### 2.14 Databases

The cBioPortal (www.cbioportal.org), Oncomine (www.oncomine.org) and Human Protein Atlas (www.proteinatlas.org) databases were used. The cBioPortal database was used to analyse SIRT5 mutations in prostate cancer and determine whether the mutations were meaningful. Oncomine and the Human Protein Atlas database were used to analyse the expression of SIRT5 in tumour cells.

### 2.15 Statistical analyses

All data were analysed using SPSS version 24.0 (Beijing, China) to perform chi‐square tests, or the Prism 5 (GraphPad, La Jolla, California, USA) software. All experiments were repeated at least three times independently under the same conditions. Values of *P* < .05 were considered statistically significant.

## 3 RESULTS

### 3.1 High expression of SIRT5 in prostate cancer cells correlates with tumour Gleason score

**FIGURE 1:**
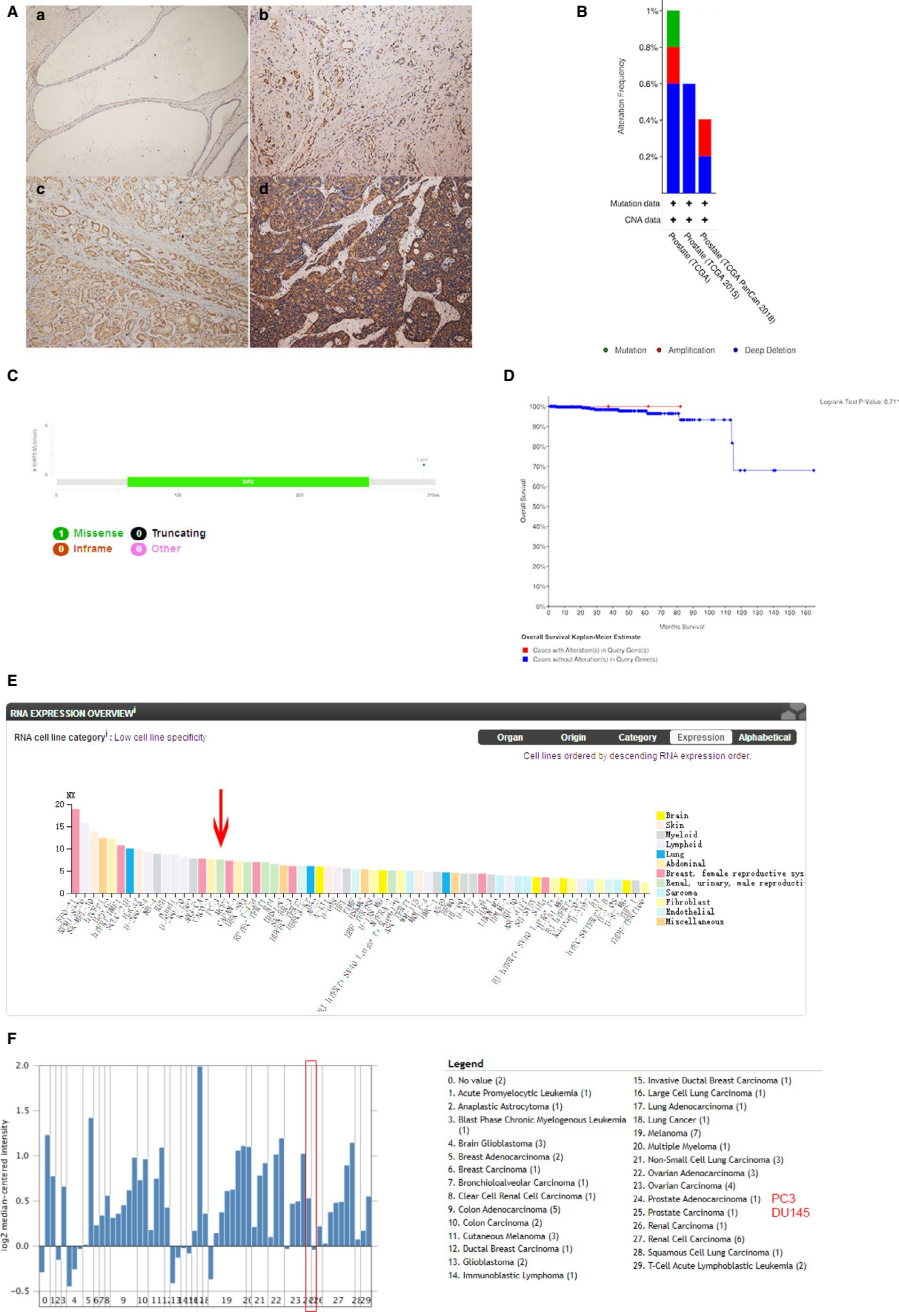
SIRT5 is highly expressed in prostate cancer tissues (A) Expression of SIRT5 protein in prostate tissue sections. (a) Normal prostate gland. (b) Prostate cancer tissue with Gleason score of 3. (c) Prostate cancer tissue with Gleason score of 4. (d) Prostate cancer tissue with Gleason score of 5. (B) Mutation type and rate of the SIRT5 gene in prostate cancer. (C) Mutation sites of the SIRT5 gene in prostate cancer; it is a L301I mutation. (D) Effect of SIRT5 gene mutations on the prognosis of prostate cancer (*P* = .771). (E) Expression of SIRT5 in representative cell lines of a variety of tumours. SIRT5 expression is high in PC‐3 cells, a representative prostate cancer cell line. (F) Same as E, expression of SIRT5 in representative cell lines of a variety of tumours. SIRT5 expression is high in PC‐3 cells, a representative prostate cancer cell line. Data were obtained from Shankavaram CellLine (Oncomine)

To study the expression of SIRT5 in human prostate cancer tissues, we performed immunohistochemical experiments on 57 randomly selected prostate tissue sections. Expression of SIRT5 was absent in normal prostate tissues but high in tumour tissues (Figure 1A). The 57 tissue sections were assigned and counted according to the Gleason score standard. When the Gleason score was equal to 7, the prostate cancer tissues could be divided into two groups: (3 + 4) and (4 + 3). The difference between the two groups was not statistically significant (*P* = .797). Similarly, the expression of SIRT5 in tissues with Gleason scores of 6 and 7 was not significantly different (*P* = .396); additionally, the expression was not significantly different in the two groups with scores of 8 and 9 (*P* = .262). However, when scores of 8 and 9 were combined as one group, SIRT5 protein expression was significantly different from that in the group with a score of 7 (*P* = .028) and the group with a score of 6 (*P* = .001) (Table 1). Therefore, we concluded that SIRT5 expression was related to the prostate cancer Gleason score.

**TABLE 1:** Expression of SIRT5 in prostate cancer tissues

	Number	SIRT5 overexpression	SIRT5 negative or weak expression	*P*‐value
Age				
≤65	30	14	16	*P* = .217
>65	27	17	10	
Gleason score				
3 + 4	6	3	3	*P* = .797
4 + 3	7	3	4	
＞7	22	18	4	*P* = .003 *R* = 0.441
≤ 7	35	13	22	
6	22	7	15	*P* = .396
7	13	6	7	
8	12	10	2	*P* = .262
9	10	8	2	
6	22	7	15	*P* = .001 *R* = 0.284
8 + 9	22	18	4	
7	13	6	7	*P* = .028 *R* = 0.371
8 + 9	22	18	4	

In the cBioPortal database, we found that the *SIRT5* gene is mutated in prostate cancer and that most of these mutations are deep deletions (Figure 1B) and missense mutations (Figure 1C). These mutations may be related to the prognosis of prostate cancer patients. However, there was no apparent statistical significance in our data (Figure 1D); consequently, we ignored the effect of *SIRT5* gene mutations on prostate cancer.

A review of the Human Protein Atlas database identified 62 cell lines of various tumours with increased SIRT5 expression. In PC‐3 cells, a representative prostate cancer cell line, this increase was considerable to other tumour cell lines (Figure 1E). A review of Shankavaram CellLine (Oncomine) the expression of *SIRT5* in PC3 cells is impressive compared to other tumour cell lines (Figure 1F).

### 3.2 SIRT5 promotes cell proliferation and migration

**FIGURE 2:**
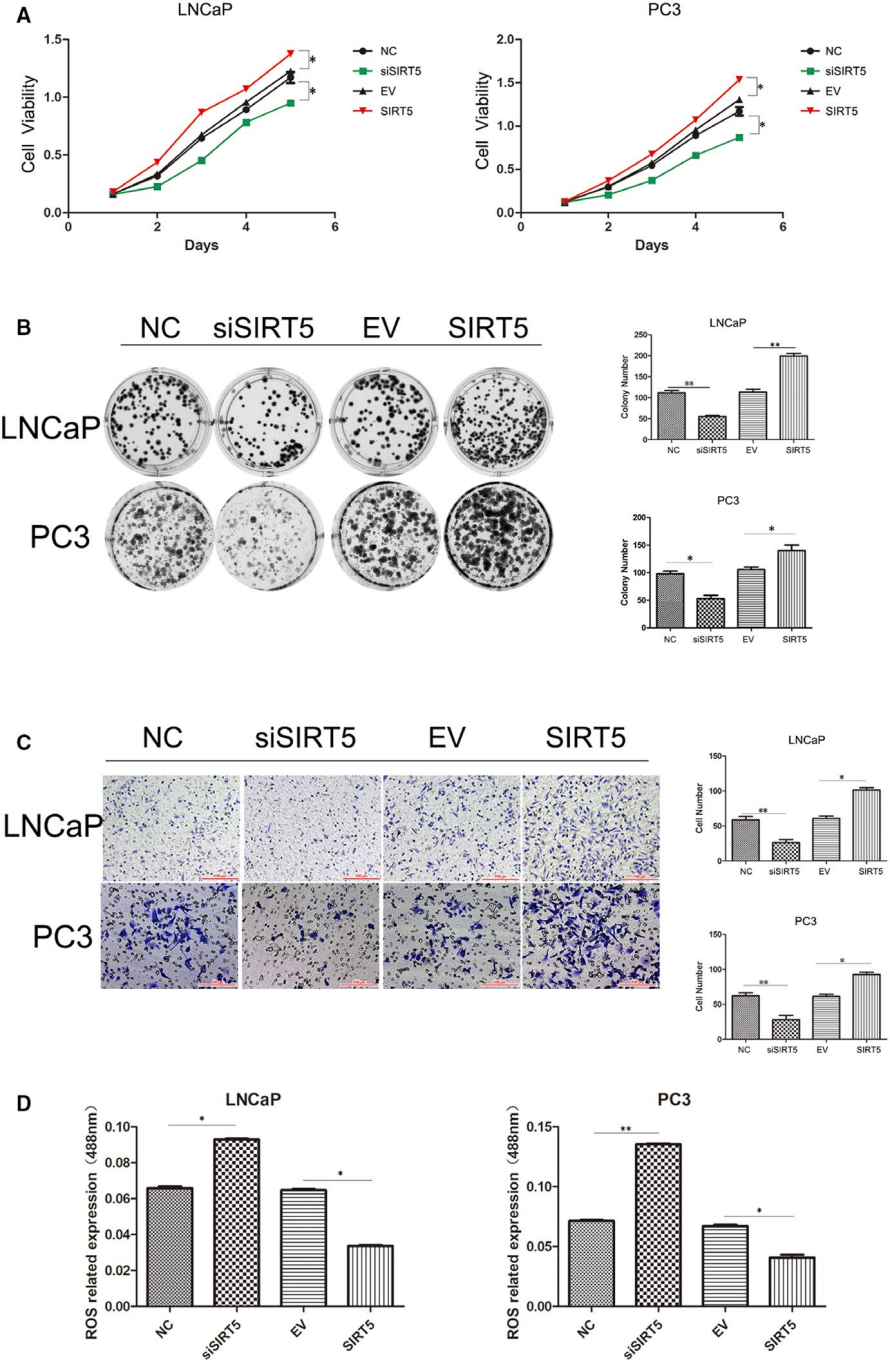
SIRT5 promotes cell proliferation and migration (A) MTS cell proliferation experiments. The effect of SIRT5 silencing or high expression on the proliferation of LNCaP (left) and PC‐3 (right) cells. *P < .05, **P < .01. (B) Colony formation experiments. After altering the expression of SIRT5, the colony forming ability of LNCaP cells and PC‐3 cells changed. The right panel shows a comparison of the number of colonies generated by these two cell lines. *P < .05, **P < .01. (C) Transwell migration experiment. After altering the expression of SIRT5, the numbers of migrating LNCaP and PC‐3 cells changed. The right panel shows a comparison of the number of migrating cells of these two cell lines. *P < .05, **P < .01. (D) High expression of SIRT5 in PC‐3 cells and LNCaP cells has the function of scavenging reactive oxygen species (ROS). *P < .05, **P < .01

Based on the above findings, we next investigated the role of SIRT5 in prostate cancer. LNCaP and PC‐3 cell lines were selected for these studies. First, it was observed that the expression of SIRT5 remained low 72 hours after transfection until the end of the experiment (Figure S1A). Cell proliferation (Figure 2A) and colony formation (Figure 2B) experiments were performed to assess the effect of SIRT5 expression on the proliferation ability of prostate cancer cells. The results of these assays in the two cell lines showed that up‐regulating SIRT5 expression promoted cell growth. Furthermore, transfection of either cell line with siRNA‐SIRT5 resulted in decreased cell proliferation and colony formation, suggesting that SIRT5 promotes cell growth.

In Transwell invasion analysis (Figure 2C), upon overexpression of SIRT5 protein in LNCaP cells, cell migration was enhanced. In addition, after interfering with SIRT5 protein expression, LNCaP cell migration ability decreased. Similar results were obtained in PC‐3 cells (Figure 2C). Addition of SIRT5 to si‐SIRT5‐treated cells restored cell behaviour (Figure S1B,C,D). Moreover, SIRT5 decreased the levels of ROS (Figure 2D). These results indicate that SIRT5 functions as a pro‐cancer factor in prostate cancer cells in vitro.

### 3.3 SIRT5 regulates the MAPK signalling pathway

**FIGURE 3:**
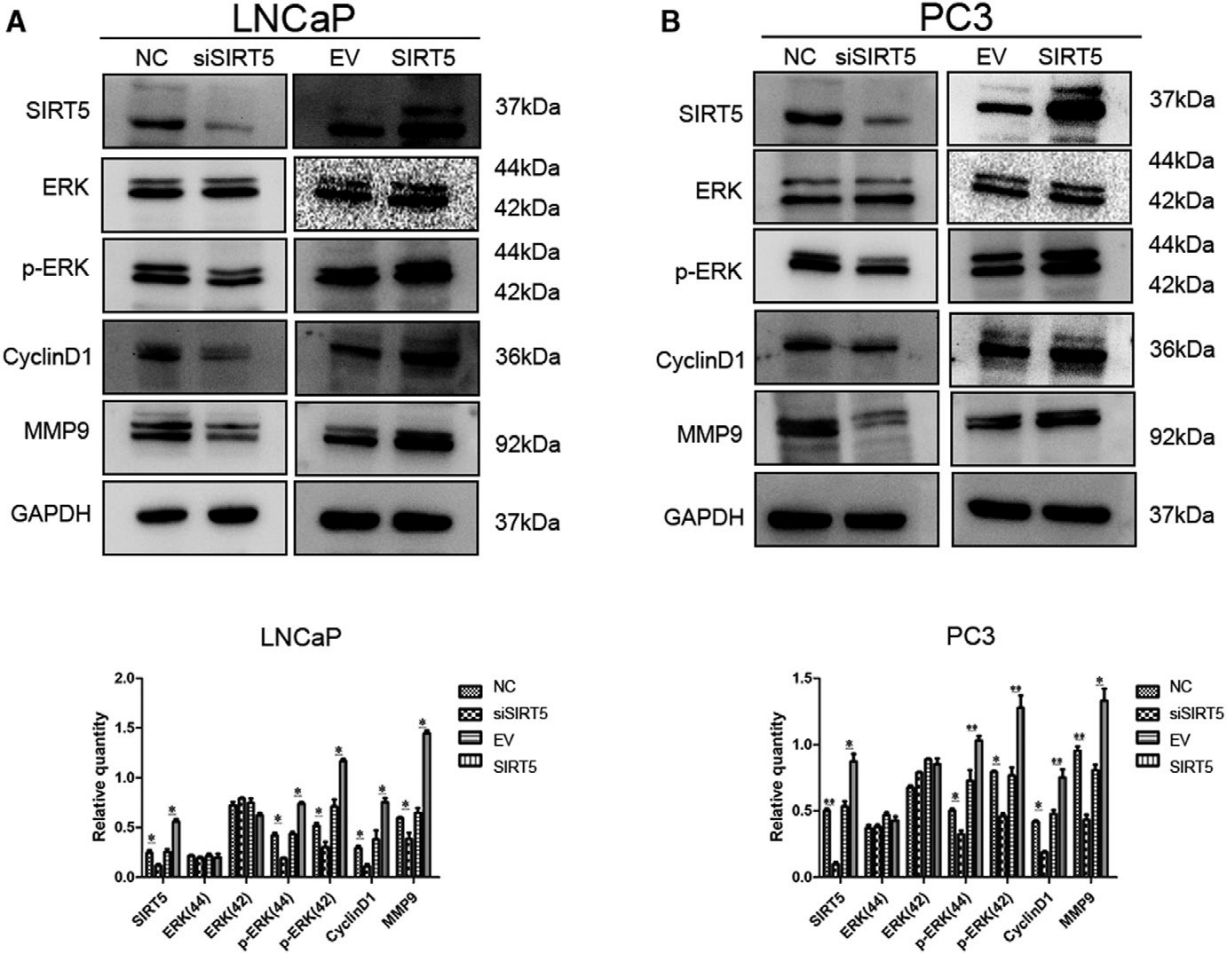
SIRT5 regulates the expression of mitogen‐activated protein kinase (MAPK) signalling proteins (A), (B) Effect of SIRT5 protein changes on the expression of functional and pathway proteins in (A) LNCaP and (B) PC‐3 cells. *P < .05, **P < .01

To understand how SIRT5 protein affects prostate cancer cells, we performed Western blot analyses. SIRT5 protein levels were modified, and the total proteins of LNCaP (Figure 3A and S2A) and PC‐3 (Figure 3B and S2B) cells were extracted. SIRT5 protein down‐regulation resulted in a significant decrease in cyclin D1 protein (related to the cell cycle) and MMP9 protein (related to migration and invasion). In addition, the activities of the MAPK pathway proteins were significantly inhibited. When SIRT5 was over‐expressed, the activity of the MAPK signalling pathway proteins and the levels of functional downstream proteins increased. These changes were consistent with the results of the in vitro proliferation and migration experiments and demonstrated that SIRT5 protein mainly regulates the MAPK signalling pathway in prostate cancer to exert tumour‐promoting effects. After increasing the expression of SIRT5 in si‐SIRT5‐treated cells, the expression of related proteins also increased (Figure S2C,D).

In order to highlight the role of SIRT5, we added a specific inhibitor of SIRT5 and found that the MAPK signalling pathway was inhibited and the expression level of related functional proteins was significantly decreased (Figure S2E,F).

### 3.4 SIRT5 combines with ACAT1 and regulates its expression

**FIGURE 4:**
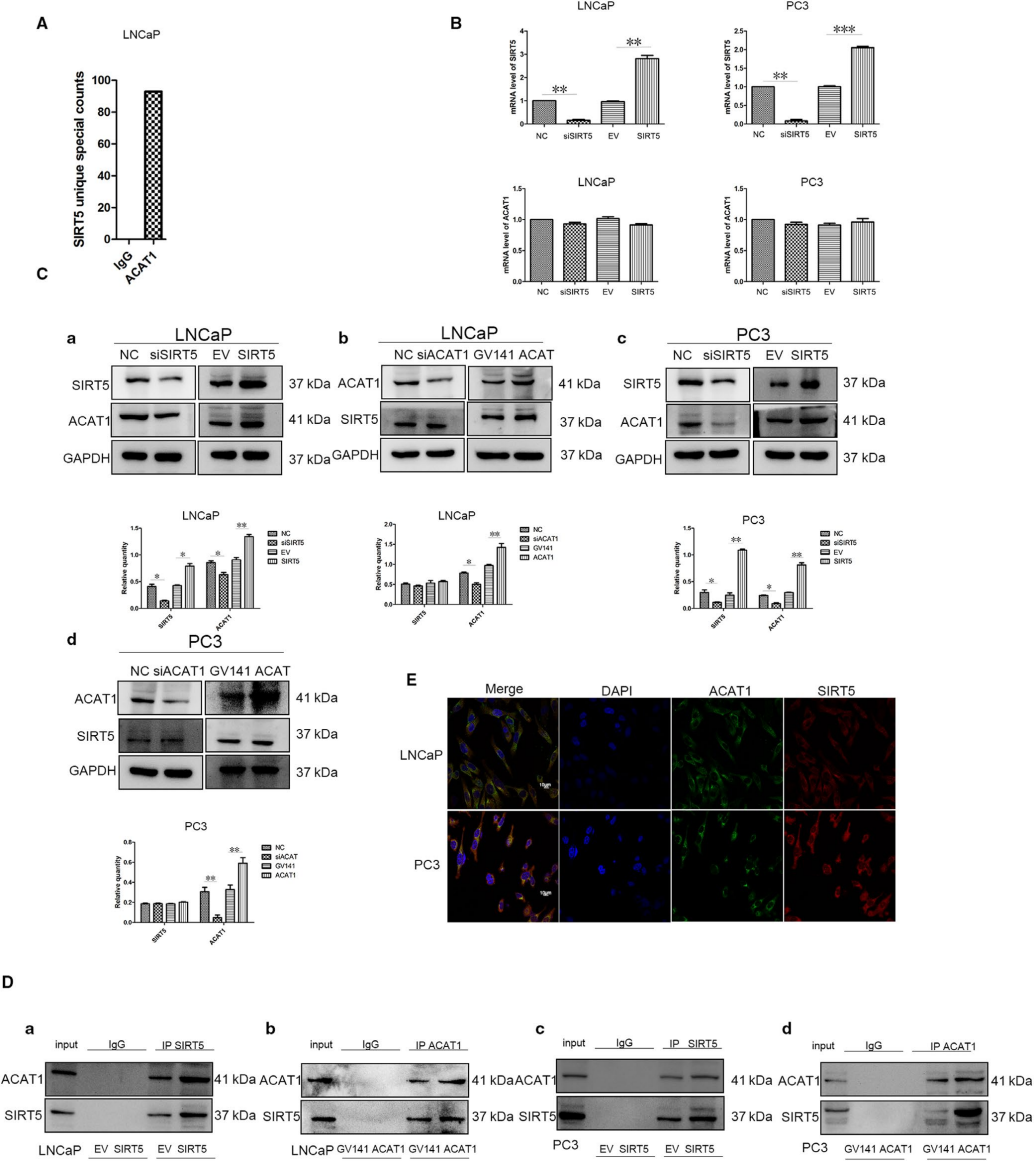
SIRT5 can combine with acetyl‐CoA acetyltransferase 1 (ACAT1) and regulate its expression (A) Mass spectrometry analysis on LNCaP cells showing that the ACAT1 protein can interact with the SIRT5 protein. (B) CAT1 mRNA levels in LNCaP and PC‐3 cells are unchanged irrespective of the SIRT5 content. *P < .05, **P < .01. (C) When the expression of SIRT5 protein is altered in (a) LNCaP and (c) PC‐3 cells, the expression of ACAT1 protein also changes. *P < .05, ** *P* < .01. When the expression of ACAT1 protein is altered in (b) LNCaP and (d) PC‐3 cells, the expression of SIRT5 protein is unchanged. (D) Quantitative co‐precipitation experiments of SIRT5 and ACAT1 proteins. (a) Transfection of SIRT5 increases its expression in LNCaP cells. (b) Transfection of ACAT1 increases its expression in LNCaP cells and co‐immunoprecipitation experiments reveal that the expression of SIRT5 protein is also increased. (c) Transfection of SIRT5 increases its expression in PC‐3 cells and co‐immunoprecipitation experiments reveal that the expression of ACAT1 protein is also increased. (d) Transfection of ACAT1 increases its expression in PC‐3 cells and co‐immunoprecipitation experiments reveal that the expression of SIRT5 protein is also increased. (E) Confocal experiments show that SIRT5 and ACAT1 proteins co‐localize in LNCaP and PC‐3 cells

To investigate how SIRT5 affected the MAPK signalling pathway, we performed mass spectrometry analyses (Figure 4A). Results showed that ACAT1 combined with SIRT5. To further investigate this finding and determine whether SIRT5 regulated ACAT1 at the gene or protein level, qRT‐PCR was performed. Irrespective of whether SIRT5 expression was increased or decreased in LNCaP cells, ACAT1 mRNA level did not change. The same result was obtained in PC‐3 cells (Figure 4B), indicating that SIRT5 does not regulate ACAT1 mRNA.

Based on these findings, we shifted our focus to protein regulation and found that after interfering with the expression of SIRT5 in LNCaP cells, the protein level of ACAT1 decreased, whereas its levels increased when SIRT5 expression was increased (Figure 4Ca). In contrast, after changing ACAT1 expression, SIRT5 level did not change (Figure 4Cb); the same results were obtained in PC‐3 cells (Figure 4Cc,D). These results indicate that SIRT5 can regulate ACAT1 at the protein level, but ACAT1 cannot regulate SIRT5 expression. However, after the expression of SIRT5 was decreased and MG‐132 was added, the expression of ACAT1 protein did not increase significantly (Figure S3A), indicating that SIRT5 does not regulate ACAT1 through the proteasome pathway.

Co‐IP experiments performed in LNCaP and PC‐3 cells showed that SIRT5 and ACAT1 combined with each other in vitro (Figure 4D). The results were confirmed through confocal experiments, which revealed that the proteins SIRT5 and ACAT1 co‐localized in the mitochondria in cells (Figure 4E and Figure S3B).

### 3.5 SIRT5 regulates the function of prostate cancer cells through ACAT1

**FIGURE 5:**
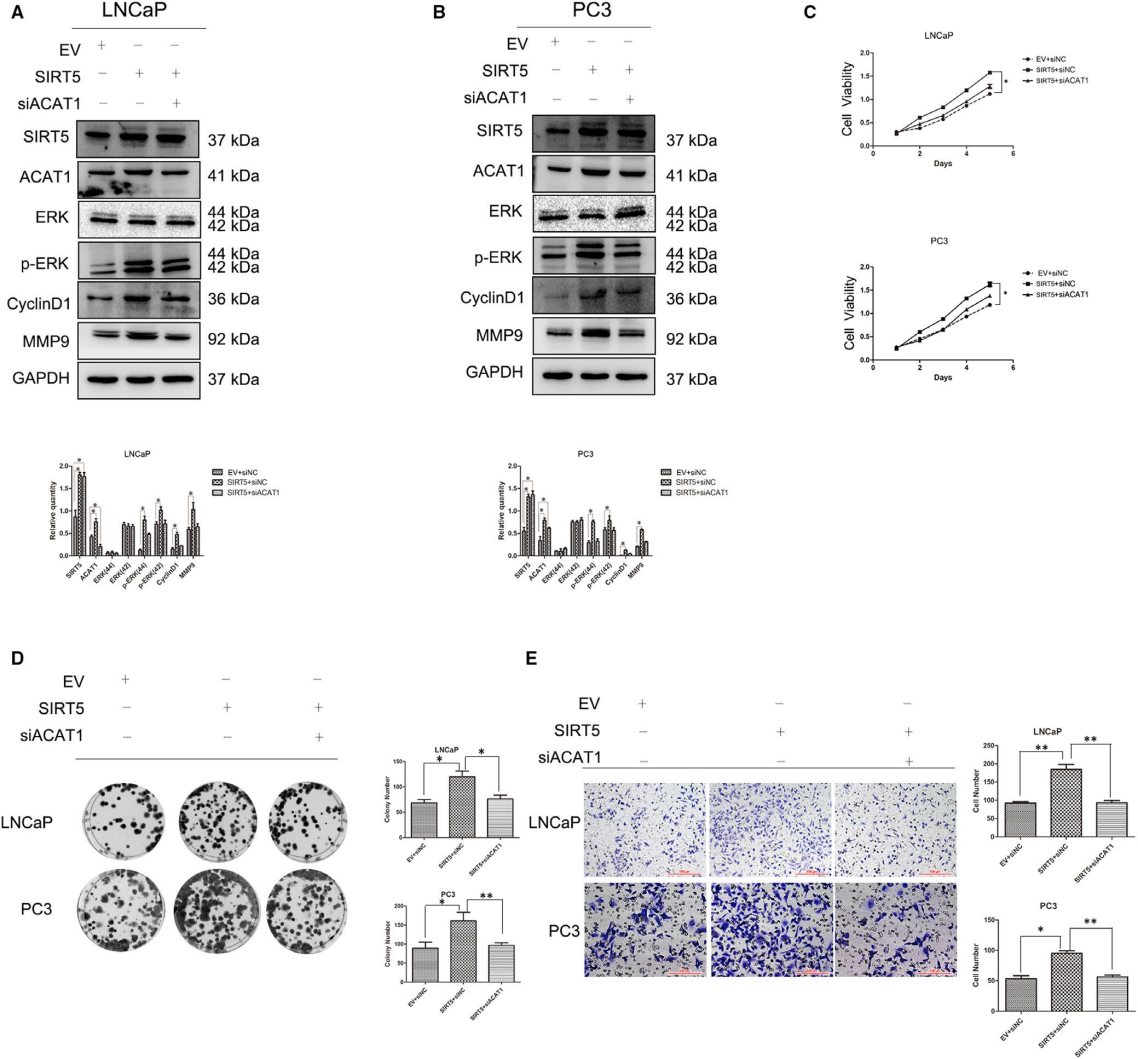
SIRT5 regulates the function of prostate cancer cells through ACAT1 Overexpression of SIRT5 and silencing of ACAT1 in (A) LNCaP cells and (B) PC‐3 cells were performed simultaneously. Changes in the expression of other proteins in these two cell lines are shown. *P < .05, **P < .01. (C) Changes of LNCaP (upper panel) and PC‐3 (lower panel) cell proliferation (MTS assay) after co‐transfection with the SIRT5 plasmid and ACAT1 siRNA. The results show that silencing ACAT1 prevents the SIRT5 tumour‐promotion effect. *P < .05, **P < .01. (D) The colony formation assay shows that the ability of SIRT5 to promote colony formation is weakened in both LNCaP and PC‐3 cells after co‐transfection of the SIRT5 plasmid and ACAT1 siRNA. The right panel shows the number of colonies generated by these two cell lines. *P < .05,**P < .01. (E) The Transwell migration assay shows that the ability of SIRT5 to promote cell migration is weakened after co‐transfection of the SIRT5 plasmid and ACAT1 siRNA in both LNCaP and PC‐3 cells. *P < .05,**P < .01

In view of the above results, we understood the effect of ACAT1 protein on prostate cancer cells. Throughout the experiment, silencing of ACAT1 protein was effective (Figure S4A,B). Under normal expression of SIRT5, silencing of ACAT1 protein can also inhibit the MAPK signalling pathway in LNCaP (Figure S4C) and PC‐3 (Figure S4D) cells. Next, we explored the relationship between these proteins and the MAPK signalling pathway in LNCaP (Figure 5A) and PC‐3 (Figure 5B) cells. Compared with that in the control group and cells transfected with flag‐SIRT5 alone, MAPK signalling pathway activity was decreased with co‐transfection of siRNA‐ACAT1 and flag‐SIRT5, consequently decreasing the expression of the downstream functional proteins Cyclin D1 and MMP9. On addition of a specific inhibitor of the ACAT1 protein, this phenomenon was more obvious (Figure S4E,F). ACAT1 silencing also inhibited the effects of SIRT5 in the MTS (Figure 5C), colony formation (Figure 5D) and Transwell migration (Figure 5E) assays. Further, when the expression of SIRT5 protein was decreased, ACAT1 rescued the expression of related proteins (Figure S4G,H).

Overall, these results demonstrate that SIRT5 regulates the MAPK signalling pathway through ACAT1 and has a tumour‐promoting role in prostate cancer.

## 4 DISCUSSION

Prostate cancer is one of the most common malignancies in men.^26^ The mortality rate in prostate cancer is related to obesity, physical exercise, smoking and antioxidants.^27^ Presently, prostate cancer treatment imposes a serious economic burden.^28^ Thus, there is an urgent need for more effective treatments to improve the survival rate of patients with this disease.

Although major advances have been made in the early detection, diagnosis and treatment of prostate cance^29^, ^30^ and various treatments have been applied to this disease, the mortality rate remains high.^29^ Therefore, it is very important to study the occurrence and development of prostate cancer as well as identify new treatment targets and methods.

Using the cBioPortal, Human Protein Atlas databases, Oncomine and immunohistochemistry experiments, we showed that SIRT5 was expressed in prostate cancer tissue and cell lines at both the mRNA and protein levels. The expression level of SIRT5 protein in tumours was markedly higher than that in normal adjacent tissues. Statistical analysis of 57 tissue sections showed that the expression of SIRT5 was related to the Gleason score of prostate cancer. This result suggested that SIRT5 might promote prostate cancer. Examination of the databases indicated SIRT5 gene mutations in prostate cancer; however, these mutations had no significance in the prognosis of the disease.

With limited clinical samples, in vitro experiments were performed first. In MTS and colony formation assays, it was evident that high expression of SIRT5 promoted proliferation and colony formation in prostate cancer cells. With transwell experiments, we showed that high expression of SIRT5 protein promoted prostate cancer cell migration.

At the protein level, results corresponding to the in vitro proliferation and migration experiments were obtained. Specifically, when SIRT5 levels were up‐regulated, Cyclin D1 (cell cycle‐related) and MMP9 (related to migration and invasion) protein expression increased. We also found that SIRT5 regulated the expression of the MAPK signalling pathway proteins. This pathway plays very important roles in tumorigenesis and development.^31^, ^32^, ^33^, ^34^

To understand how SIRT5 regulated the MAPK signalling pathway, mass spectrometry analysis was performed, which identified ACAT1 as a potential substrate of SIRT5. Through mRNA, protein, immunofluorescence and Co‐IP experiments, we found that SIRT5 can bind to ACAT1 and regulate its expression, but that SIRT5 does not regulate ACAT1 protein expression through the proteasome pathway. SIRT5 protein in mitochondria may regulate ACAT1 by regulating the level of autophagy, but this needs to be verified in future studies.

We determined that ACAT1 can regulate the MAPK signalling pathway; therefore, we believe that SIRT5 regulates the MAPK signalling pathway through ACAT1, thereby promoting prostate cancer. MAPK protein is not only present in the nucleus and cytoplasm but may also be present in the mitochondria.^35^ However, the mechanisms through which SIRT5 and ACAT1 affect the MAPK signalling pathway and by which the MAPK protein shuttles between the mitochondria, cytoplasm and nucleus remain to be studied further. Nevertheless, we can confirm that the SIRT5‐ACAT1 axis plays a very important role in prostate cancer. In addition, this axis may help develop new treatment strategies for prostate cancer, which may provide new hope for the treatment of this disease.

## CONFLICT OF INTEREST

The authors have no conflict of interest to declare.

## AUTHOR CONTRIBUTIONS

**Jingqian Guan:** Data curation (equal); Software (equal); Writing‐original draft (equal); Writing‐review & editing (equal). **Xizi Jiang:** Data curation (equal); Software (equal). **Junda Gai:** Data curation (equal); Software (equal). **Xiaodan Sun:** Data curation (equal); Software (equal). **Jinming Zhao:** Data curation (equal); Software (equal). **Ji Li:** Data curation (equal); Software (equal). **Yizhuo Li:** Data curation (equal); Software (equal). **Ming Cheng:** Data curation (equal); Software (equal). **Tengjiao Du:** Data curation (equal); Software (equal). **Lin Fu:** Data curation (equal); Funding acquisition (supporting); Software (equal); Writing‐review & editing (equal). **Qingchang Li:** Funding acquisition (lead); Methodology (equal); Writing‐original draft (equal); Writing‐review & editing (equal).

## ETHICS APPROVAL AND CONSENT TO PARTICIPATE

This study was approved by the Ethics Committee of China Medical University.

## Supporting information

Supplementary Material
