# Supplementary material for: Sirtuin 5 regulates the proliferation, invasion and migration of prostate cancer cells through acetyl‐CoA acetyltransferase 1
Source: J Cell Mol Med. 2020 Oct 26;24(23):14039–49. doi: 10.1111/jcmm.16016 (PMC7753991; doi:10.1111/jcmm.16016)
Supplement: Supplementary file 1 — Supplementary Material [file JCMM-24-14039-s001.docx]

**Supporting information**

**
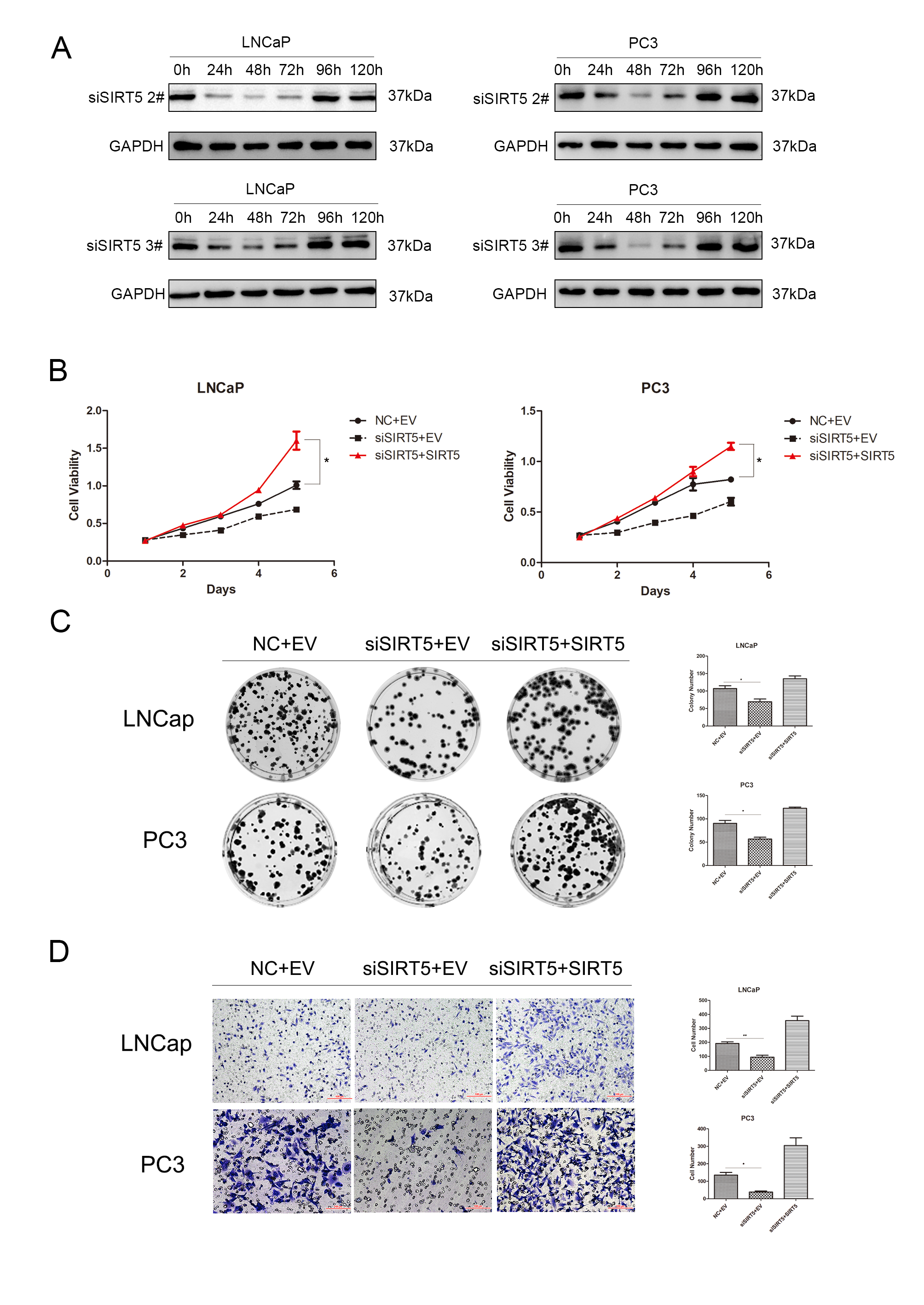
**

**Figure 1**

1. The low expression of SIRT5 persists within 72 h after conversion.
2. MTS cell proliferation experiments. The effect of SIRT5 silencing on the proliferation of LNCaP (left) and PC-3 (right) cells. *P < 0.05, **P < 0.01.
3. Colony formation experiments. After altering the expression of SIRT5, the colony formation ability of LNCaP cells and PC-3 cells changed. The right panel shows a comparison of the number of colonies generated by these two cell lines. *P < 0.05, **P < 0.01.
4. Transwell migration experiment. After altering the expression of SIRT5, the numbers of migrating LNCaP and PC-3 cells were changed. The right panel shows a comparison of the number of migrating cells of these two cell lines. *P < 0.05, **P < 0.01.


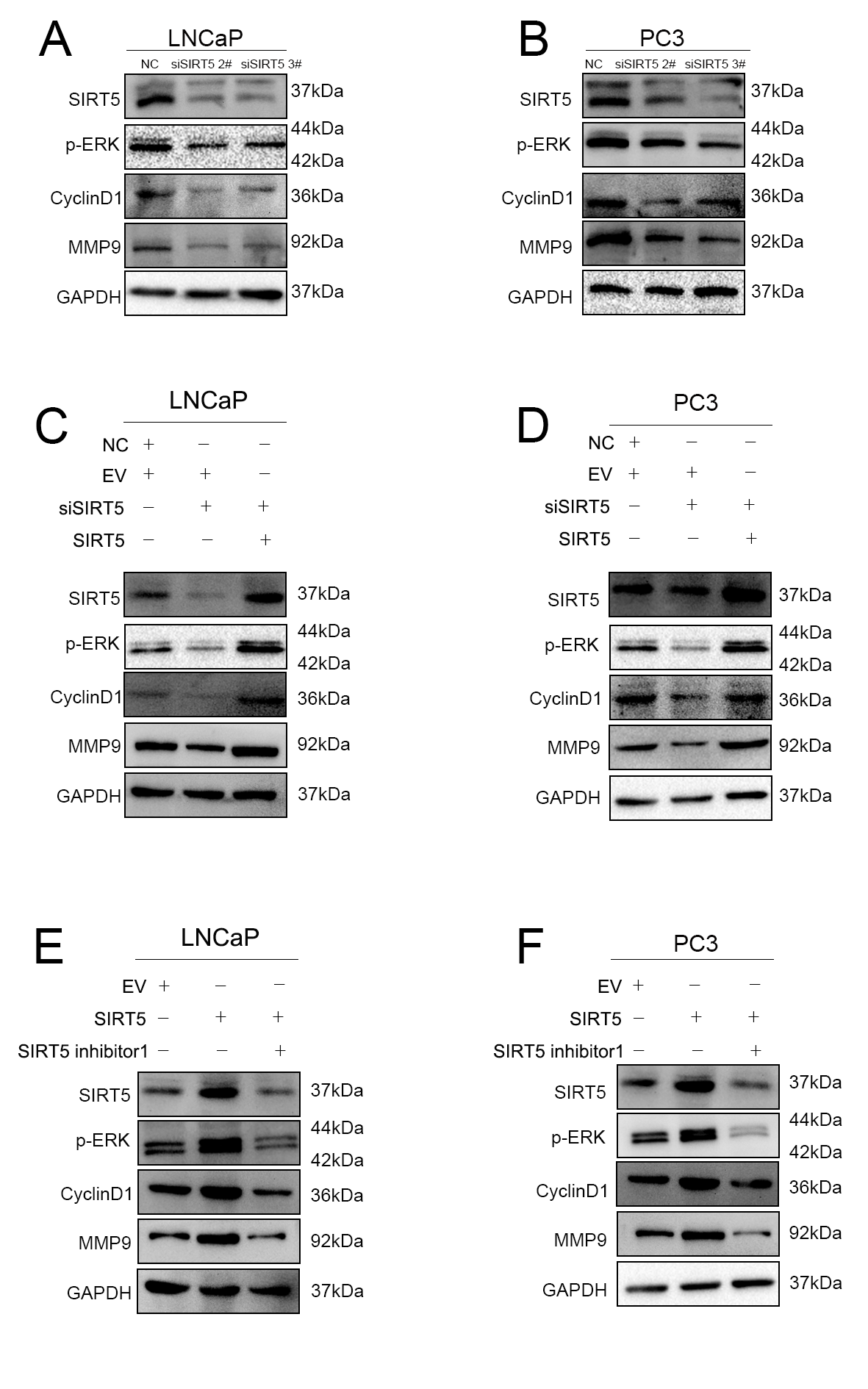


**Figure 2**

A, B. Effect of SIRT5 protein changes on the expression of functional and pathway proteins in (A) LNCaP and (B) PC-3 cells. *P < 0.05, **P < 0.01.

C, D. Further addition of SIRT5 to si-SIRT5-treated (C) LNCaP and (D) PC-3 cells showed that related protein levels were rescued. *P < 0.05, **P < 0.01.

E, F. The expression of related proteins after adding a specific inhibitor of SIRT5 to (E) LNCaP and (F) PC-3 cells. *P < 0.05, **P < 0.01.


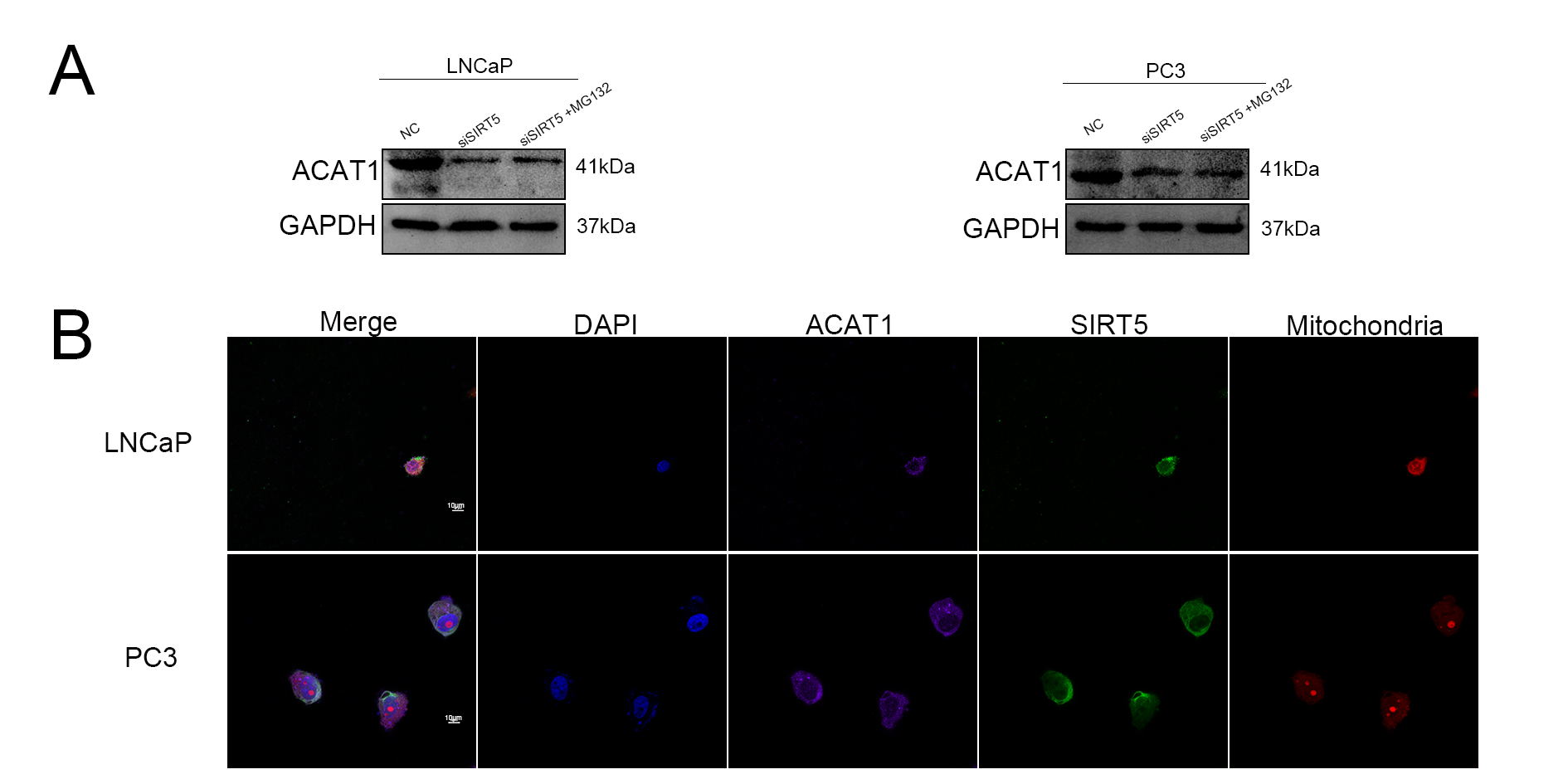


**Figure 3**

1. In the case of SIRT5 protein silencing, the proteasome inhibitor MG-132 does not stabilize the expression of ACAT1 protein.
2. Confocal experiments show that SIRT5 and ACAT1 proteins co-localize in the mitochondria in LNCaP and PC-3 cells.


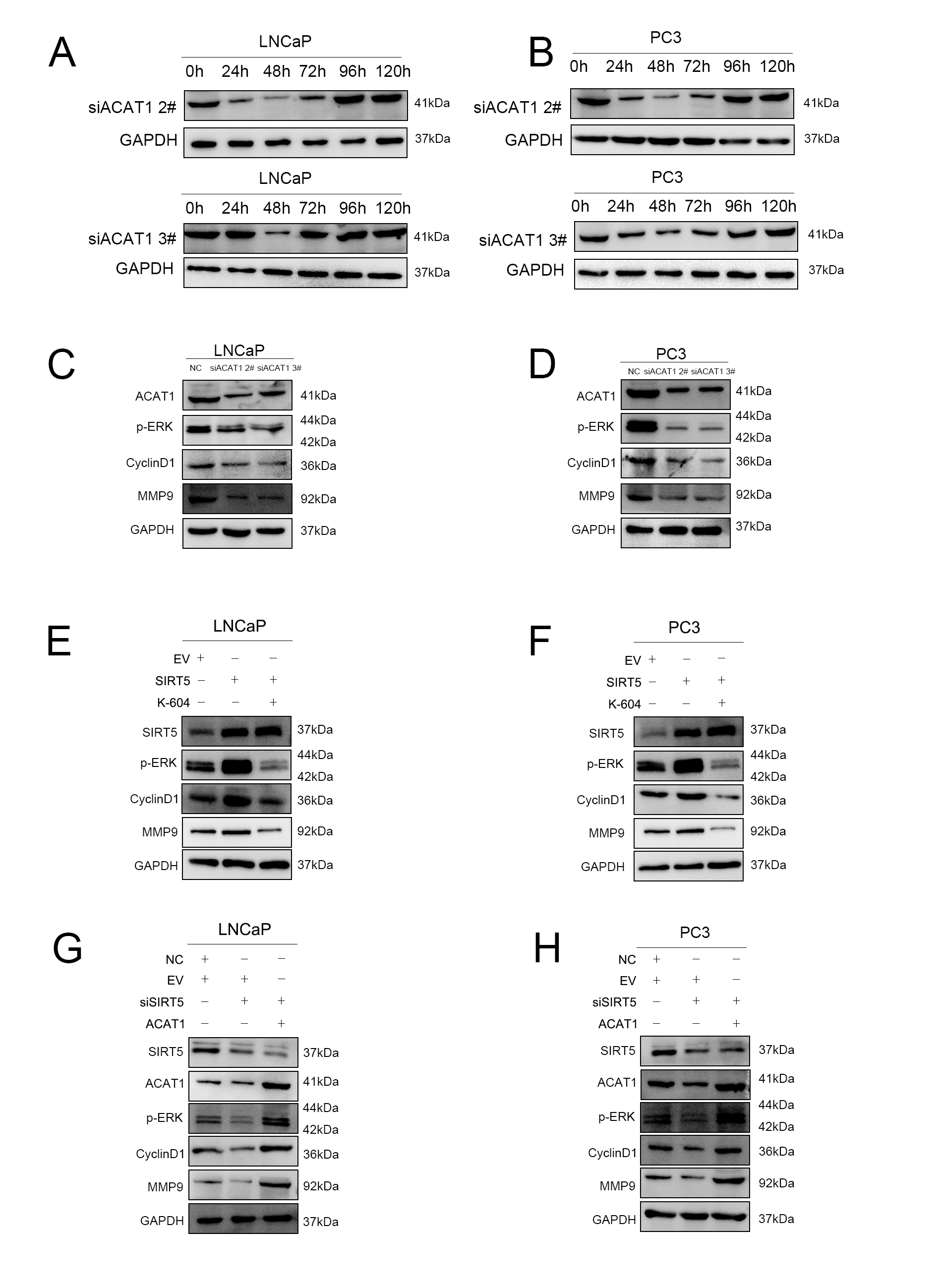


**Figure 4**

1. The low expression of ACAT1 persists for 72 h after transfection with si-ACAT1.

B,C. Effect of ACAT1 protein changes on the expression of functional and pathway proteins in (B) LNCaP and (C) PC-3 cells. *P < 0.05, **P < 0.01.

D,E. The changes of related proteins after adding ACAT1 specific inhibitor to (D) LNCaP cells and (E) PC-3 cells with high expression of SIRT5. *P < 0.05, **P < 0.01.

F,G. In (F) LNCaP cells and (G) PC-3 cells, ACAT1 can reverse the impact of SIRT5 silencing. *P < 0.05, **P < 0.01.
